# The Orthopædic Hospital at Shepherd's Bush

**Published:** 1922-04

**Authors:** 


### THE ORTHOPÆDIC HOSPITAL AT SHEPHERD'S BUSH.

It is a hopeful sign to read in "The Times," of March 15, that the Hammersmith Guardians propose to have a conference with the Ministry of Pensions in order to find the lines of a settlement of the melancholy dispute which has arisen over the question of rent. It is clear that the national interest, and a due consideration for the large number of ex-Service patients involved, make it imperative that an amicable settlement should be reached, and that the magnificent work which is being done at Shepherd's Bush should continue. It is equally clear that it was not a useful contribution towards such a settlement that the Minister of Pensions in the House of Commons should imply that he was being "blackmailed" by the Guardians; and in the conference which, it may be hoped, will have been held with happy results before these lines appear, such an unfortunate expression will, no doubt, have been withdrawn.

The Hammersmith Guardians have a perfectly good claim to an economic rent. In its absence they would, in effect, be bearing an undue share of what is clearly a national burden. The suggestion of arbitration made by Dr. Acland in "The Times" would be a perfectly simple and satisfactory means of settling the matter if agreement could not otherwise be reached on such an issue as the question of what is the amount of the economic rent.

We say "a melancholy dispute" advisedly. It must be supposed that unless Sir Robert Jones had drawn attention to the question in his very moderately worded letter to "The Times" on March 2, the Pensions Ministry would have gone forward with their project of transferring the in-patients from Shepherd's Bush to hutments in Richmond Park, and opening one or more out-patient departments elsewhere. It was not to be supposed that Sir Robert Jones—to whose inspiration and pioneer work the splendidly equipped institution at Shepherd's Bush is due—could witness such a calamitous procedure without entering a public protest.

Of the work of the hospital he says:—

"For its unique equipment and admirable staff work, Shepherd's Bush as a pioneer of recent orthopædic surgery and for its continued post-war activity, has become recognised not only by the medical world as a distinguished centre, but by the men themselves, wherever they may be, as a city of refuge when hope of recovery grows faint. To have carried on this curative work in the past and to complete it in the future, equipment and staff organisation must be of the highest possible standard."

Dr. Acland supplemented this, in a letter on the following day, by some details of the work done, from which it is sufficient to quote that, during 1921, 3,905 officers and men were under treatment as in-patients, and that there were 101,596 out-patient attendances; and in addition 297 cases daily treated in the departments for hydrotherapy. Everyone, says Dr. Acland, would recognise the need that both parties to the dispute should practise legitimate economies, but "no one will forgive them if they practise these economies at the expense of men who have suffered, and still are suffering, so much in the service of their country." Sir Robert Jones had already given several cogent reasons why the hospital could not be transplanted without materially affecting the welfare of the pensioner.

There is a danger at the present time that, in the blessed name of economy, positive damage is going to be inflicted on the community. It is difficult, indeed, to conceive in the Shepherd's Bush case that real economy is going to be effected, even if sentimental considerations were ignored. Removal expenses, duplicate equipment, loss of time of skilled staff, lack of co-ordination of treatment between out-patient and in-patient services, prolongation of treatment because of inferior facilities—all these things involve extra outlay, which might well swamp the sum of £2,000 per annum remaining in dispute.

As stated above, this particular question happily seems to be in a fair way to getting settled satisfactorily. But we must be continually on our guard, and ready to raise our voices in vigorous protest against the sacrifice to the "urgent necessity of economy" of still more urgent matters of health.

---

Mr. Robert Heath, of Biddulph Grange, has offered his house and grounds to the North Staffordshire Cripples' Aid Society, to be used as an orthopædic hospital for North Staffordshire. The question of the heavy cost of maintenance is being considered, and the various public health authorities have been asked to what extent they would be likely to avail themselves of the proposed new hospital, which, if supported, could greatly extend the Society's work.

